# Supplementary material for: Identifying subgroups of high-need, high-cost, chronically ill patients in primary care: A latent class analysis
Source: PLoS One. 2020 Jan 29;15(1):e0228103. doi: 10.1371/journal.pone.0228103 (PMC6988945; doi:10.1371/journal.pone.0228103)
Supplement: S1 File — (DOCX) [file pone.0228103.s001.docx]

Qualitative persona of class 1 ‘older adults living with partner’

Mr. Taylor is 74 years old and living together with his wife, who receives pension benefits as well. For a couple of years now, he is suffering from multimorbidity (i.e. diabetes in combination with COPD).

Qualitative persona of class 2 ‘older adults living alone’

Mrs. Williams is 79 years old and living alone. Her husband has passed away five years ago. For some time now, Mrs. Williams has to deal with visual disorders and osteoarthritis. In addition, she has been suffering from diabetes for a long time.

Due to severe mood disorders, she is dependent on sickness benefits. Besides the mood disorders, she already suffers from asthma since her youth. For a couple of years, she receives care from a specialized mental health professional, alongside the care she receives from the GP.

Qualitative persona of class 3 ‘middle-aged, employed adults with family’

Mrs. Jones is 51 years old and living together with her husband and two sons of 19 and 22 years old. She works parttime as nursing assistant in a nursing home. Mrs. Jones has diabetes and chronic back and neck disorders.

Due to severe mood disorders, she is dependent on sickness benefits. Besides the mood disorders, she already suffers from asthma since her youth. For a couple of years, she receives care from a specialized mental health professional, alongside the care she receives from the GP.

Qualitative persona of class 4 ‘middle-aged adults with social welfare dependency’

Ms. Smith is 52 years old and living alone for some time now. Due to severe mood disorders, she is dependent on sickness benefits. Besides the mood disorders, she has been suffering from asthma since her youth.
